# Whither Coronabonds? The Past and Future of the EMU in the Coronavirus Pandemic

**DOI:** 10.1007/s10272-020-0887-z

**Published:** 2020-06-07

**Authors:** Bodo Herzog

**Affiliations:** ESB Business School, Economics, Macroeconomics, Hochschule Reutlingen, Reutlingen University, Alteburgstraße 150, 72762 Reutlingen, Germany

## Abstract

The long-term fiscal and economic damage of eurobonds in a rule-based fiscal architecture — as history corroborates — would be greater than the historical challenge of the coronavirus pandemic, unless there is a political union in Europe.

The European sovereign debt crisis is almost history, yet the next challenge is looming. The novel coronavirus, medically known as SARS-CoV-2, is sending shockwaves into the real economy worldwide. The global economic downturn is a new burden for the still weak eurozone. In February and March 2020, the European Central Bank (ECB) boldly adopted monetary measures in line with the 'whatever-it-takes' paradigm. At the moment, one is reminded of the phrase, "If the euro fails, then Europe fails" by German chancellor Angela Merkel.

In terms of a unified fiscal stimulus, economists and politicians have found the Holy Grail, too: eurobonds, also known as coronabonds, European Safe Bonds or a fiscal capacity (Blanchard et al., 2017; Brunnermaier, 2017; Bénassy-Quéré et al., 2018). Yet, this debate betrays a certain naivety as there is a lack of meticulous analysis of the conditions in the eurozone despite the need for Keynesian deficit spending in a crisis.

What is the root cause and what is the symptom? Can eurobonds solve the problem or do they merely treat the symptom?

## Status quo: Social and economic life in standstill

The coronavirus pandemic originated in the Chinese city of Wuhan at the beginning of December 2019. By mid-February 2020, the epidemiological spread reached Europe. As it has evolved into a global outbreak, the World Health Organization (WHO) declared the coronavirus (COVID-19) a pandemic on 11 March 2020. The disease has brought social and economic life in China, Europe and much of the world to a standstill. Entire countries are in lockdown or have imposed a quarantine. This is necessary as there are currently no medicines or vaccines available. Social distancing and domestic isolation are the only ways to slow the spread of the virus.

However, the necessary health protection measures have a massive impact on the economy and financial markets. On Monday and Thursday, 9 and 12 March 2020 respectively, stock markets worldwide plummeted by 10% (Figure 1). The price of oil fell by 30% as the global demand for crude oil collapsed amid the pandemic.

The developments in the financial markets foreshadow what will become visible in the real economy soon. Supply chains from China have been disrupted. Initial estimates suggest that China is experiencing the deepest recession in decades. So are Europe and the US (German Council of Economic Experts, 2020; Wollmershäuser, 2020). The coronavirus crisis could take on unprecedented proportions in economic history, despite the fact that politicians are intervening with financial aid programmes and central banks are increasing liquidity.

## Corona crisis management in Europe

The coronavirus pandemic reveals once again the incompleteness of the European Union. National borders were closed without any coordination. Additionally, the pandemic has exposed major country-specific differences in the health care systems. Health policy however, just like fiscal and tax policy, is the responsibility of each member state. Thus, the country-specific spending preferences are not reflecting a lack of European solidarity, but rather conscious or neglected decisions in the EU member states.

It is true that the economic shock demands a European and a global response (García Herrero, 2020). The first priority is rapid action via monetary policy in parallel with fiscal expansion. Indeed, both monetary and fiscal policies switched to an expansionary path in the eurozone in March 2020. Nevertheless, it is still questionable whether the simultaneous demand and supply shock can be cushioned. In this respect, the call for further fiscal instruments, especially in the weakening eurozone, is understandable.

Figure 1
**Coronavirus stock market crash**

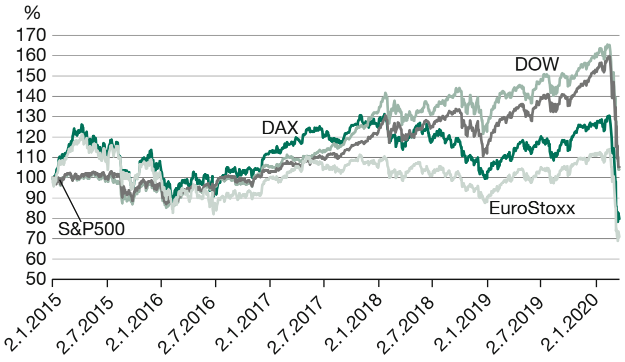

Source: Author's own illustration based on Data FactSet.

However, the debate is dominated by fear and panic. But fear is a bad advisor. The widespread paralysis is irrational, as is evident during terrorist attacks (e.g. 9/11) or natural disasters. The current type of shock can cause panic because we suddenly expect that many people will die in a short period or experience an economic bankruptcy.

The fiscal challenges of individual member states in the eurozone are negligible against the background of COVID-19, which is a life-threatening situation. But the economic challenges cannot be ignored either. A strong health care system needs financial resources and therefore a strong economy. However, the present panic leads to uncontrollable behavioural reactions such as the herd-like behaviour seen in fire sales on the stock markets as well as hoarding and panic purchases of groceries. This creates a typical run-like situation, as people are afraid of going away empty-handed, similar to bank runs in financial crises.[1]

The continuous expansion of financial liquidity for the past 20 years is the fallout of several crises, including the 'new economy' bubble, the terrorist attacks of 11 September 2001, the global financial crisis of 2008-2009 and the Eu-

1 Interestingly, the consequences of irrational fears or panic are often deadlier in modern societies. For example, a study by Gaissmaier and Gigerenzer (2012) shows that after the terrorist attack of 9/11, many Americans avoided airplanes and switched to cars. As a result, in the year following the attack, there were 1,600 more road deaths than average.

Figure 2
**European Central Bank balance sheet**
in billion euro

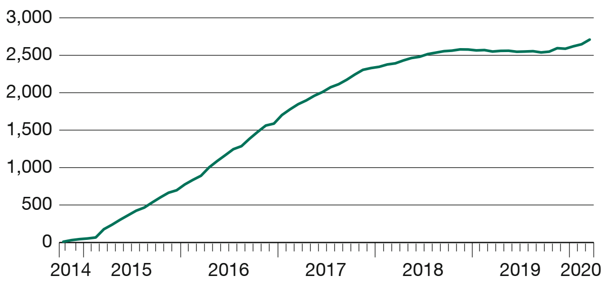

Source: Author's own illustration based on ECB Statistical Data Warehouse.

ropean sovereign debt crisis. Globally and in the eurozone, central banks have expanded their balance sheets on an unprecedented scale (Figure 2). Although the measures might be right at the moment, their long-term social and economic effects remain unexplored in research so far. Anecdotal evidence shows a misallocation of capital, overconsumption, a growing income inequality, lower happiness and higher stress levels in Western countries (Case and Deaton, 2017; Alvaredo, 2018).

## Debating coronabonds

Coronabonds are an instrument to jointly issue public debt across all member states of the eurozone. Economists and politicians, regardless of their ideology, support this idea if the debate is academically transparent and reveals all preconditions. Yet, there is a predominant cacophony of proponents and opponents who in some cases politely omit or deliberately conceal the required preconditions.

First, the EU or the eurozone is not a state. The Treaty on the Functioning of the European Union (TFEU) clearly states this. There is no European fiscal sovereignty and hence no right to issue public debt. Moreover, there is no fiscal budget and no euro finance minister with cut-through clauses at member state levels. Fiscal policy as well as social, labour and health policies are the responsibility solely of member states. This explains the country-specific differences in the tax burden, retirement age and level, number of hospital beds or the level of general public services. These differences are reflecting country-specific political preferences.

Secondly, despite the notion of being 'united in diversity', centralised instruments will not solve the structural differences of eurozone member states, as sovereignty and country-specific preferences remain national. For instance, the German Federal Constitutional Court (BVerfG, 1993) has repeatedly emphasised that the core principle of the eurozone is the 'stability community' (BVerfG, 1993). To that extent, eurobonds raise the constitutional question of intensified budgetary control with rights of intervention at the level of member states (Calliess, 2016). However, the budgetary law is one of the identity-determining tasks of member states as defined by the German Basic Law in the Lisbon judgment. Fiscal sovereignty is particularly relevant to democracy, and it is assigned to the environment of the eternity clause of Article 79(3) of the Basic Law (BVerfG, 2020). Thus, the President of the German Constitutional Court already signalled some time ago that the idea of eurobonds touches on sensitive areas of the Basic Law (Voßkuhle, 2010). In other words, coronabonds may be difficult to implement in the eurozone due to both national and European legal constraints.

Thirdly, the historical lessons of failed monetary unions are being ignored due to the acute pandemic in Europe. The breakdown of other historical supranational monetary unions, such as the Scandinavian Monetary Union, the Latin Monetary Union and the Austria-Hungarian Monetary Union, should be a wake-up call to all during times of high uncertainty. Indeed, all historical monetary unions have failed not because of too little but too much solidarity, as the principle of liability and control was undermined over time. In the end, a huge collapse occurred as some member states became more indebted and sought to externalise the cost of public debts to the others (Theurl, 1996; Bordo and Jonung, 1999). In short, moral hazard is not an abstract risk, but a reality in a supranational monetary union – even within the eurozone (Berthold et al., 2014).

A large body of academic literature documents moral hazard risks in the eurozone and recommends precautions, whether strict fiscal rules, such as the Maastricht Treaty, or a complete transfer of sovereignty, i.e. a political union (Beetsma and Uhlig, 1999; Beetsma and Bovenberg, 1999, 2000, 2003; Herzog, 2018b). With a political union in place, the instrument of joint public borrowing via eurobonds would be fully endorsed. On the contrary, the medium-term damage of an institutional mixture – as history demonstrates – could act as a catalyst for the break-up of the eurozone. Hence, how realistic is a political union? Where is the European roadmap for a political union from France, Italy and Spain? How quickly can it be implemented – in one, five, or 50 years?

In the absence of a political union and in light of historical lessons, the founding fathers of the Maastricht Treaty designed the eurozone with a rules-based fiscal architecture

(Herzog and Choi, 2017). Within this framework, there can be no joint borrowing through eurobonds, as the mixed structure touches on the principle of liability and control and generates instability (Herzog, 2012; Herzog and Ferencz, 2019).

The present rules-based fiscal architecture is built on the pillars of the no-bailout rule in Article 125 TFEU, the prohibition of debt financing by the ECB in Article 123 TFEU and the Stability and Growth Pact. The fiscal architecture was enhanced in the aftermath of the global financial crisis. Indeed, the fiscal framework was supplemented by new regulations, including the European Semester and the Two- and Six-pack (Herzog, 2018a). These instruments guarantee liability and control.

Fourthly, when considering the idea of a European fiscal equalisation scheme, it is important to note that such schemes do not work well even on a small scale, i.e. on the national level (Konrad, 2014). Furthermore, a financial equalisation scheme cannot be simply transferred to the European level (Herzog, 2018b). Even if eurobonds were appropriate to alleviate the fragility of the eurozone, the legal and political prerequisite must be in place: a political union.

The German Council of Economic Experts (2005) pointed out that the fiscal equalisation system in Germany cannot solve the structural heterogeneity across the *Länder* or federal states. Despite fiscal equalisation and a federal budget, the differences between the *Länder* or between US states are even greater than between the member states of the eurozone. On the other hand, all national fiscal equalisation schemes have tough cut-through powers at the state level. For example, there are intervention rights in ultima ratio, including the appointment of a 'restructuring commissioner' in federal states that do not comply with the rules. This commissioner could raise taxes and reduce state debts. This would considerably restrict state sovereignty. If such a mechanism is even necessary in a single country, such as the US or Germany, it would be equally urgent in a heterogeneous group of countries. Yet, without a political union this mechanism is a profound illusion.

## Roadmap to the future

As long as the premises for a closer fiscal integration are not taken into consideration, the debate about eurobonds will remain marked by European naivety. Solidarity is not a one-way street (Herzog, 2012). No economist can seriously claim that the eurozone is an optimal currency area, according to the theory (Mundell, 1961; Herzog and Hengstermann, 2013). Thus, no one can expect eurobonds to solve the structural problems of the eurozone. Imposing eurobonds without the prerequisites would only destabilise the eurozone.

Politicians proposing this instrument would first have to show whether they are willing to hand over sovereignty to Brussels in order to establish a political union. It remains highly unlikely that countries such as Italy, Spain or France would be willing to transfer all sovereignty in fiscal, tax, labour, health and social policies to Brussels. But that is indispensable for issuing joint debt by eurobonds or a eurozone fiscal capacity.

The eurozone is probably decades away from this epochal step. Against this background, present crisis management must make use of existing institutional mechanisms. Under certain circumstances, additional instruments could be added, provided that liability and control remain in one hand (Calliess, 2015). In any case, the fiscal potential of eurobonds is not more powerful than the hitherto untapped combination of assistance from the European Stability Mechanism (ESM) in conjunction with the ECB's Outright Monetary Transactions (OMT) programme.

In regard to crisis prevention, however, the existing fiscal architecture needs to be made more binding as long as policymakers do not establish a political union. The aim is to ensure that all, not only the role models, stick to European fiscal rules. Accordingly, it is conceivable that rule enforcement needs to be strengthened by partial cut-through powers, especially in the event of incompliance (Herzog, 2013; Calliess, 2018). In addition, monetary penalties should be reconsidered by a vote-and-reputation-function in case of incompliance (Herzog and Hengstermann, 2013). It is also possible to supplement the ESM with a debt restructuring mechanism (Herzog, 2017).

The EU and eurozone is a voluntary union of democratic states, structured as a 'stability community' according to the philosophy of the Lisbon Treaty. Thus, each member state must support the common rules. Eurobonds destroy the general principle of liability and control of the eurozone – even as an exceptional instrument in an unprecedented pandemic. The long-term fiscal and economic damage of eurobonds in a rule-based fiscal architecture – as history corroborates – would be greater than the historical challenge of the coronavirus pandemic, unless there is a political union in Europe.
